# Cellular Stress and Immune Activation in Celiac Disease: Is the Chaperone System a Key Player?

**DOI:** 10.3390/biology15100805

**Published:** 2026-05-19

**Authors:** Giuseppe Vergilio, Giusy Vultaggio, Rosalia Gagliardo, Letizia Paladino, Francesca Rappa

**Affiliations:** 1 Department of Biomedicine, Neuroscience and Advanced Diagnostics, University of Palermo, 90127 Palermo, Italy; giuseppe.vergilio@unipa.it (G.V.); francesca.rappa@unipa.it (F.R.); 2 Institute of Translational Pharmacology, National Research Council of Italy (CNR), 90127 Palermo, Italy; rosaliapaola.gagliardo@cnr.it; 3 Department of Theoretical and Applied Sciences, eCampus University, 22060 Novedrate, Italy; letizia.paladino@uniecampus.it

**Keywords:** celiac disease, heat shock protein 60 (Hsp60), epithelial stress, Toll-like receptors (TLRs), innate immune activation

## Abstract

Celiac disease (CD) is a chronic immune-mediated enteropathy triggered by the ingestion of gluten in genetically predisposed individuals. While the adaptive immune response to deamidated gliadin peptides represents a central pathogenic mechanism, growing evidence suggests that epithelial stress and innate immune activation play a fundamental role in the onset and persistence of the disease. Heat shock proteins (Hsps), central regulators of cellular proteostasis, have emerged as potential mediators at the interface between epithelial distress and immune signaling. This review discusses the involvement of major Hsp families, including Hsp27, Hsp60, Hsp70, and Hsp90, in the pathophysiology of CD. The altered expression of Hsp27 and Hsp70 in the intestinal mucosa reflects a persistent state of epithelial stress that often persists despite a strict gluten-free diet (GFD). We focus specifically on Hsp60, whose extracellular release under stress conditions may allow it to function as a damage-associated molecular pattern (DAMP), engaging Toll-like receptors and promoting NF-κB- and inflammasome-dependent inflammatory pathways. Although direct mechanistic evidence linking Hsp60 to CD remains limited, the convergence of epithelial stress signs, Toll-like receptor (TLR) upregulation, and prolonged innate immune activation supports the hypothesis of a stress-induced inflammatory amplification circuit in the coeliac mucosa. Further studies are essential to clarify the pathogenic relevance and potential therapeutic implications of this proposed axis.

## Simple Summary

Celiac disease (CD) is an immune-mediated disorder triggered by gluten in genetically predisposed individuals. Beyond the adaptive immune response, epithelial stress and innate immunity play key roles in disease onset and persistence. Heat shock proteins (HSPs), especially Hsp27, Hsp60, Hsp70, and Hsp90, may link epithelial damage to immune activation. Hsp60, in particular, may act as a damage signal (DAMP), activating inflammatory pathways through Toll-like receptors. Although evidence is still limited, these mechanisms suggest a stress-driven amplification of inflammation in CD, highlighting the need for further research.

## 1. Introduction

### 1.1. Autoimmune Diseases

Autoimmune diseases (ADs) are complex, multifactorial disorders resulting from a delicate interplay between genetic susceptibility, epigenetic modifications, and environmental triggers [1]. This alteration leads to the inability of B and T lymphocytes to correctly distinguish autoantigens from non-autoantigens, promoting the activation of immune responses directed against the body’s own tissues [2]. The loss of immunological tolerance is a central mechanism in autoimmunity and is associated with an HLA-dependent genetic predisposition [3]. Current estimates place the global prevalence of autoimmune disease at 3–5%, although incidence varies between different conditions [3]. The role of genetic factors is evidenced by the high concordance observed in twins and first-degree relatives compared to non-biological relatives sharing the same environment [4]. However, identifying causal variants and understanding their functional effects remains complex [4]. At the same time, diverse environmental factors contribute to the onset of autoimmunity. Bacterial and viral infections act as immunological triggers, while exposures such as cigarette smoking, toxic substances, and dietary factors can modulate risk through epigenetic mechanisms [5,6]. Among these disorders, celiac disease (CD) is one of the most common conditions [7,8].

### 1.2. Celiac Disease: Prevalence, Pathogenesis, Diagnosis and Treatment

#### 1.2.1. Prevalence and Epidemiology

Numerous epidemiological studies have demonstrated that CD was historically underdiagnosed and that its global distribution reflects wheat consumption and human migrations. Around 10,000 years ago, in the “Fertile Crescent,” favorable environmental conditions allowed the cultivation of wheat and barley. This shift enabled the settlement of nomadic populations and triggered migrations that spread gluten-containing cereals across the Mediterranean and into Europe, contributing to a shared genetic background between these populations [8].

Celiac disease is now recognized as one of the most common genetic disorders, with a global prevalence of approximately 1%. This percentage fluctuates between 0.5% and 1% in Europe and North America, reaching 2% to 3% in Finland and Sweden [9]. Furthermore, CD shows a higher incidence in women, with a male to female ratio of 1:2.8 [8].

#### 1.2.2. Pathogenesis

CD is a chronic enteropathy triggered by gluten ingestion in genetically predisposed individuals [8]. Gluten, present in wheat, barley, rye and spelt, consists of prolamins and glutelins [8]. Prolamins, rich in glutamine, are partially resistant to gastrointestinal digestion. Peptides derived from gliadin represent optimal substrates for deamidation by tissue transglutaminase (tTG) [8]. In individuals carrying the HLA-DQ2 and/or HLA-DQ8 haplotypes, deamidated peptides acquire a high affinity for HLA molecules, promoting the activation of CD4^+^ T lymphocytes and an aberrant immune response [8,9,10]. The resulting production of pro-inflammatory cytokines, together with the activation of cytotoxic intraepithelial lymphocytes (IELs), induces progressive mucosal damage, manifesting as crypt hyperplasia and villous atrophy [8,11,12]. This structural remodeling compromises the absorptive surface, leading to nutrient malabsorption [8,13]. Beyond genetics, factors such as early gluten introduction, viral infections, and gut microbiota alterations influence disease onset. Increased intestinal permeability, mediated by zonulin, further facilitates the passage of immunogenic peptides [14,15].

#### 1.2.3. Diagnosis

Diagnosis relies on a combination of serological tests and histological evaluation. The detection of anti-tissue transglutaminase (anti-tTG) and anti-endomysium antibodies (EMAs) serves as the first level of screening due to high sensitivity [8]. Duodenal biopsy remains the gold standard, revealing histological changes categorized by the Marsh classification [16]: Marsh I (increased IELs), Marsh II (crypt hyperplasia), and Marsh III (villous atrophy). This morphological progression reflects the cytotoxic activity of IELs and the intensity of epithelial damage [8,13].

#### 1.2.4. Treatments

Currently, no approved pharmacological therapies exist; the only effective treatment is a strict, life-long gluten-free diet (GFD), which usually resolves symptoms and mucosal damage [8]. Several emerging strategies are under investigation, including prolyl endopeptidases (PEPs) to hydrolyze gliadin, tTG2 inhibitors; and HLA-DQ blockers [8]. However, the persistence of mucosal stress signals even during a strict GFD suggests that alternative pathogenic pathways remain active.

#### 1.2.5. Aims and Methodology of the Review

This narrative review integrates classic knowledge of CD with emerging evidence on the chaperone system, specifically the Hsp60-TLR4 axis. To ensure a rigorous and balanced synthesis, a structured search was conducted across PubMed, Scopus and Web of Science databases, covering literature published between 2000 and 2026. The search employed keywords including “heat shock proteins”, “Hsp60”, “celiac disease”, and “Toll-like receptors”.

We opted for a narrative approach rather than a systematic or scoping review because the evidence regarding the Hsp60-TLR4 axis in CD is currently exploratory and stems from highly heterogeneous experimental models (ranging from in vitro signaling to human histological studies). Such heterogeneity precludes a formal meta-analysis but allows for a broader, cross-disciplinary integration of findings, which is essential for proposing a new pathogenic framework.

Unlike previous general overviews, this work uniquely identifies the Hsp60-TLR4 axis as a functional bridge between epithelial stress and innate immune activation, moving beyond a purely descriptive approach to offer a novel conceptual model for persistent mucosal inflammation.

### 1.3. The Chaperone System

To understand how the intestine copes with such chronic aggression, we must look at the chaperone system (CS). This sophisticated cellular network, comprising molecular chaperones, co-chaperones, and cofactors, is the primary guardian of protein homeostasis (proteostasis) [13]. It ensures that proteins are correctly folded, transported, and assembled or safely degraded if damaged. When CS fails, proteostasis collapses, paving the way for a broad spectrum of pathologies [17,18].

The most prominent members of this system are the heat shock proteins (Hsps), an evolutionarily conserved group of proteins expressed both constitutively and in response to stress stimuli (Table 1) [19,20].

Hsps perform fundamental functions in proper protein folding, prevention of protein aggregation, and regulation of intracellular protein trafficking, contributing significantly to the maintenance of cellular homeostasis and proteostasis [36,37]. Hsp expression can be constitutive or inducible [38]. Certain members of the Hsp family exhibit constitutive expression under physiological conditions to maintain routine cellular functions, others are rapidly upregulated in response to different forms of cellular stress. This response arises from a variety of stimuli including heat stress, hypoxia, and exposure to toxic agents or heavy metals, as well as inflammatory processes and metabolic alterations [20]. In the gut, epithelial cells are under constant pressure from environmental stressors that often elicit a massive Hsp response. Increasingly, evidence suggests that Hsps are not just passive markers of stress, but they are active modulators of the immune system. Their involvement is well-established across a broad spectrum of immune and chronic inflammatory conditions, including rheumatoid arthritis [39], diabetes mellitus [40], myasthenia gravis [41], and inflammatory bowel disease (IBD) [42]. In such contexts, Hsps may bridge the gap between cellular distress and chronic immune activation, either by attempting to restore balance or, in certain instances, by acting as endogenous signals that perpetuate the inflammatory cycle.

## 2. Heat Shock Proteins and Epithelial Stress in Celiac Disease

Current evidence suggests that multiple Hsps follow a convergent pattern of dysregulation in CD. This altered expression is tightly coupled to a state of intestinal epithelial stress that may precede or even persist independently of overt mucosal inflammation. Although individual Hsps differ in their subcellular localization and specific biological roles, their collective imbalance reflects a condition of chronic epithelial distress. This persistent state potentially lowers the threshold for immune-mediated damage, thereby predisposing individuals to both the onset and accelerated progression of the disease.

### 2.1. Hsp27

Heat shock protein 27 (Hsp27), a member of the small Hsps family, is a key guardian against proteotoxic stress, acting as a molecular chaperone to prevent protein aggregation [43]. Beyond cytoprotection, Hsp27 regulates apoptosis by inhibiting mitochondrial cytochrome c release and modulating pro-apoptotic signaling [44]. It also stabilizes the cytoskeleton, helping to preserve the integrity of the epithelial barrier. In the context of CD, Hsp27 has emerged as an early marker of epithelial stress. Immunohistochemical studies have demonstrated overexpression in enterocytes even before the onset of overt inflammation or villous atrophy [45]. Remarkably, first-degree relatives of celiac patients exhibit increased Hsp27 levels; despite lacking an adaptive immune response to gluten and maintaining a normal mucosal architecture, these individuals display clear signs of subclinical epithelial distress [45]. Since Hsp27 overexpression characterizes active CD and persists in a significant proportion of patients on a GFD, it suggests that epithelial stress may not be merely a secondary effect of gluten, but rather an intrinsic feature of the predisposed epithelium. Take together, these findings place Hsp27 at the center of the interaction between epithelial stress and immune response, where its persistent alteration may lower the threshold for damage in genetically susceptible individuals.

### 2.2. Hsp60

Hsp60 (chaperonin 60, Cpn60) is a highly conserved mitochondrial chaperone that, in cooperation with Hsp10, ensures the correct folding of newly synthesized proteins while maintaining mitochondrial proteostasis [46]. Under conditions of stress or injury, Hsp60 can accumulate in the cytosol or translocate to the extracellular space. In this extracellular compartment, it acts as a danger-associated molecular pattern (DAMP), triggering both innate and adaptive immune responses. Dysregulation of Hsp60 is often associated with mitochondrial dysfunction, increased oxidative stress, and the perpetuation of chronic inflammation [46]. In IBD, for instance, Hsp60 expression significantly increases in the inflamed colonic mucosa of patients with Crohn’s disease and ulcerative colitis. Its aberrant cytoplasmic localization suggests an active role in driving mucosal immune activation [29]. While direct evidence linking Hsp60 to the CD pathogenesis is currently limited, the established roles of oxidative stress and mitochondrial distress in gluten-induced damage make Hsp60 a highly plausible player. This hypothesis warrants further targeted experimental investigation to bridge the gap between mitochondrial health and celiac immunity.

### 2.3. Hsp70

Heat shock protein 70 (Hsp70) belongs to a family of ATP-dependent chaperones central to protein quality control. It promotes proper protein folding, prevents aggregation, and facilitates the degradation of damaged proteins [47]. Beyond these roles, Hsp70 interferes with caspase-dependent apoptosis and modulates the immune response when released extracellularly during tissue damage [47]. In CD, Hsp70 serves as a marker of intestinal oxidative stress. Research by Piątek-Guziewicz et al. reported significant Hsp70 overexpression in the duodenal mucosa of adult celiac patients, both in untreated subjects and in patients on a GFD [48]. The persistence of elevated Hsp70 levels, despite clinical and serological remission, suggests that a GFD may not completely normalize the underlying epithelial distress. While Hsp70 acts as an adaptive response to reactive oxygen species, potentially preserving barrier integrity through its anti-apoptotic effects, its chronic elevation correlates with persistent histopathological alterations in adults [48]. This highlights a clear dissociation between clinical recovery and true mucosal healing. Consequently, Hsp70 may also represent a more sensitive biomarker than conventional serology for detecting residual mucosal activity or intermittent gluten exposure [48].

### 2.4. Hsp90

Heat shock protein 90 (Hsp90) is a cytosolic chaperone essential for the stability and activity of client proteins, including kinases and pro-inflammatory transcription factors [42]. Through this function, Hsp90 directly regulates inflammatory pathways. In IBD, Hsp90 expression increases significantly in the inflamed mucosa and only partially reduces after therapy [32]. Its levels correlate positively with CD4^+^ T-cell infiltration, suggesting it actively modulates local immunity rather than just responding to damage [32]. Pharmacological inhibition of Hsp90 effectively attenuates colitis in animal models by reducing pro-inflammatory cytokine production and modulating regulatory T-cell activity [42]. As with Hsp60, direct evidence in CD remains scarce. However, since CD features intense CD4^+^ T cell activation and persistent epithelial stress, the role of Hsp90 in stabilizing immune signaling is an area of great interest. This knowledge gap identifies Hsp90 as a prime candidate for future investigation into the mechanisms of gluten-related pathology.

## 3. Hsp60–TLR Interplay in Celiac Disease

**Figure 1:**
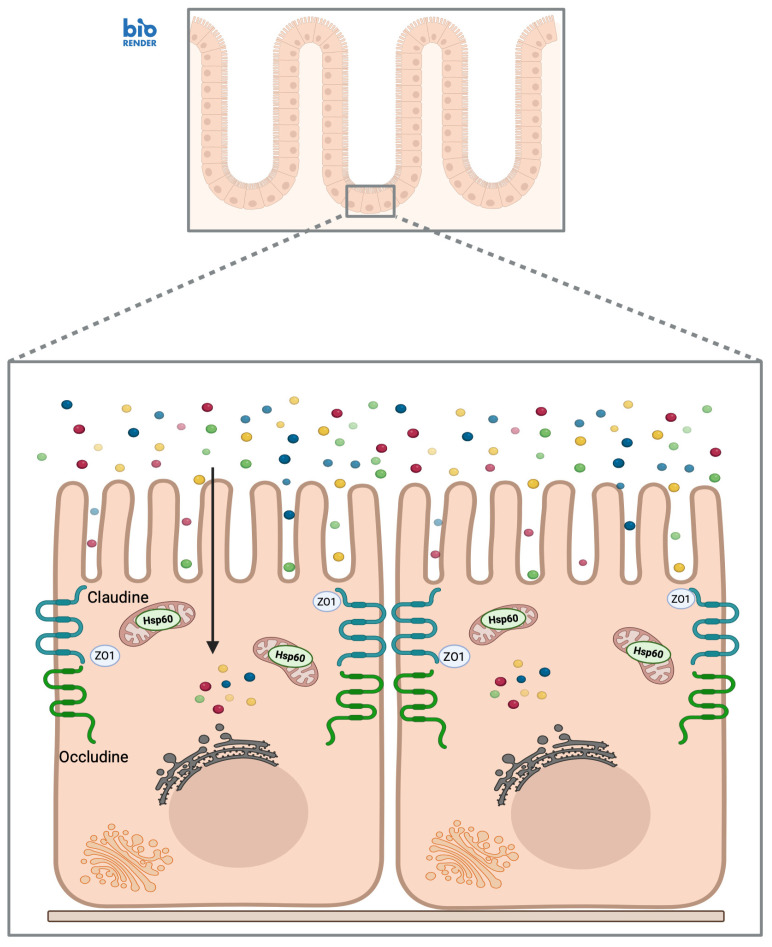
The chaperone system in healthy mucosa. The small intestinal mucosa features well-organized villi and a preserved epithelial architecture. Enterocytes maintain proper polarity and are anchored by intact tight junctions (TJs). These junctions, composed of structural proteins such as claudins, occludin, and ZO-1, ensure selective paracellular permeability and a robust barrier. Under physiological conditions, Hsp60 resides primarily within mitochondria, where it exerts its canonical chaperone function by assisting protein folding and supporting cellular homeostasis. Created in BioRender. Bucchieri, F. (2026) https://BioRender.com/t8e6siq, accessed on 4 February 2026.

CD features a chronically inflammatory mucosal microenvironment where persistent epithelial stress and the continuous activation of innate and adaptive immunity interact. In this delicate balance, endogenous molecules released in response to cellular damage may acquire unconventional immunological functions, potentially contributing to the perpetuation of inflammation [49,50]. As widely reported in the literature, Hsp60 emerges as a potential mediator hypothesized to link intracellular distress to the activation of the mucosa immune circuit [51]. Under physiological conditions, Hsp60 primarily resides within the mitochondria, where it ensures proper protein folding and mitochondrial homeostasis (Figure 1) [51].

**Figure 2:**
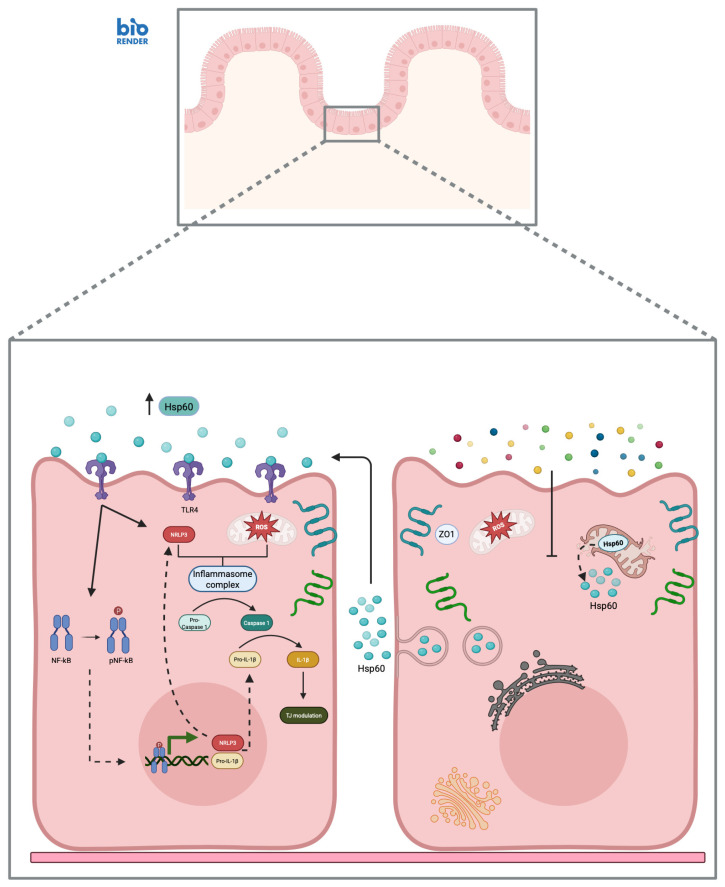
Hsp60-TLR4 axis and inflammatory signaling in celiac mucosa. The celiac mucosa exhibits villous atrophy, characterized by marked shortening and architectural distortion of the epithelial layer. Environmental triggers, such as gliadin-derived peptides, induce cellular stress, driving Hsp60 overexpression and its subsequent release into the extracellular space. In this context, extracellular Hsp60 acts as a damage-associated molecular pattern (DAMP), binding to Toll-like receptors (TLRs), particularly TLR4, expressed on enterocytes. This interaction triggers intracellular signaling cascades that promote NF-κB phosphorylation and nuclear translocation, resulting in transcriptional upregulation of pro-inflammatory genes, including NLRP3 and pro-IL-1β. Concurrently, increased ROS production contributes to the activation of the NLRP3 inflammasome, enabling caspase-1 to cleave pro-IL-1β into its mature form. Once secreted, mature IL-1β exerts autocrine and paracrine effects on tight junctions, causing structural alterations that increase paracellular permeability. The resulting barrier dysfunction facilitates enhanced antigen translocation, thereby perpetuating mucosal inflammation. Created in BioRender. Bucchieri, F. (2026) https://BioRender.com/t8e6siq, accessed on 4 February 2026.

However, under conditions of inflammatory stress or structural damage to the intestinal epithelium, Hsp60 can accumulate in the cytosol or enter the extracellular space (Figure 2) [52].

In this context, the protein is thought to act as a DAMP, engaging innate immunity receptors, specifically Toll-like receptors 4 (TLR4) [52]. This interaction triggers pro-inflammatory signaling pathways, such as p38 MAPK and NF-kB, leading to the induction of cytokines like IL-1β, TNF-α and IL-6. Furthermore, TLR4 activation promotes the production of reactive oxygen species (ROS), which serve as key signals for NLRP3 inflammasome activation and subsequent IL-1β release [53,54,55,56]. IL-1β plays a pivotal role in the gut; beyond amplifying the inflammatory response, it directly impacts the epithelial barrier. Through the activation of myosin light chain kinase (MLCK), this cytokine induces alterations in the tight junction architecture, potentially compromising barrier integrity and increasing intestinal permeability. This breakdown could facilitate the passage of immunogenic gluten peptides, reinforcing a vicious cycle of mucosal immune activation [57,58,59]. While much of this mechanistic evidence is derived from general cellular models, the core molecular players, TLR4, NFkB, ROS, NLRP3 and IL-1β, are highly conserved components of the intestinal epithelium. Clinical studies on duodenal biopsies have demonstrated a significant upregulation of TLR2 and TLR4 in patients with active CD; notably, this overexpression often persists even in individuals adhering to a long-term GFD. Such findings suggest that innate immune activation may remain “smoldering” regardless of acute gluten exposure, leading to a hypothesized state of chronic inflammatory priming [60]. Within this primed environment, the role of exogenous triggers is well-established: peptides derived from gliadin and other wheat components, such as α-amylase/trypsin inhibitors, can directly activate TLR-dependent pathways, particularly through TLR2 and TLR4 [61]. However, in a landscape where these receptors are already upregulated, the presence of endogenous DAMPs like Hsp60 could potentially amplify the inflammatory cascade [62]. Taken together, these observations support the hypothesis that the Hsp60-TLR axis may represent a crucial intersection between epithelial stress and innate immune activation in CD. In this model, Hsp60 is proposed to function as an endogenous “distress signal” born from cellular damage, capable of translating metabolic and epithelial strain into an active inflammatory response. This mechanism may contribute to the self-perpetuating mucosal environment characteristic of the disease. Although direct functional evidence of Hsp60–TLR interaction specifically within the celiac context is still emerging, the convergence of persistent epithelial stress, TLR overexpression, and inflammasome activation points toward a powerful inflammatory amplification circuit that warrants urgent further investigation.

## 4. Discussion

The evidence examined in this review outlines a scenario in which heat shock proteins (Hsps) are not merely a consequence of mucosal damage in celiac disease (CD) but may instead represent active, early components of its pathogenesis. The mechanistic similarities between CD and inflammatory bowel diseases (IBDs) suggest that Hsps constitute a common denominator in chronic inflammatory intestinal disorders. Specifically, the persistent alteration of Hsp27, Hsp70, Hsp60, and Hsp90 suggests a state of chronic epithelial distress that may precede the adaptive immune response and persist despite clinical remission induced by a gluten-free diet (GFD) [45,48]. The overexpression of Hsp27 in enterocytes of patients with active CD identifies this protein as a sensitive marker of subclinical stress [45]. Its presence in genetically predisposed individuals without evident histological damage suggests that gluten exposure acts on already vulnerable tissue, where Hsp27 may influence disease susceptibility through its dual role as a chaperone and apoptosis regulator [41,44]. Similarly, elevated Hsp70 levels in adults in apparent remission reveal a dissociation between the normalization of conventional histological parameters and the persistence of functional mucosal alterations [48]. This indicates that oxidative stress may not fully resolve with a GFD, positioning Hsp70 as a potentially more sensitive biomarker than Hsp27 for monitoring complete mucosal healing. Furthermore, the interaction between Hsp60 and TLR4 establishes a self-sustaining pathogenic circuit [49,50,51]. By acting as a DAMP, Hsp60 engages TLR4, which is chronically overexpressed in the celiac mucosa, linking epithelial damage directly to innate immune activation [60]. The subsequent activation of NF-κB and NLRP3 pathways, resulting in IL-1β production, promotes increased intestinal permeability via MLCK-mediated damage to tight junctions [57,58,59]. This creates a vicious cycle where inflammation and structural damage mutually reinforce each other [62]. Despite these promising findings, a significant research gap persists regarding the clinical application of Hsps. Currently, chaperones are not utilized in clinical settings for the diagnosis, monitoring, or treatment of CD. The primary bottleneck for this clinical translation is not the technical availability of assays; indeed, standardized, high-throughput methods such as the ELISA and automated immunoblotting are widely accessible for measuring both intracellular and extracellular Hsps. However, the real challenge lies in the lack of disease-specificity and the absence of validated clinical cut-offs for CD. Since Hsps are universal markers of cellular distress, their levels are also elevated in other conditions such as IBD, intestinal infections, and food allergies. Therefore, a critical gap remains in distinguishing CD-specific Hsp signatures from those of other enteropathies. From a therapeutic perspective, although the Hsp60-TLR4 axis represents a “druggable” target, the challenge lies in developing gut-specific modulators that avoid interfering with the essential cytoprotective functions of chaperones in healthy tissues.

Future research should prioritize longitudinal cohort studies and the use of CD-specific experimental models such as 3D intestinal organoids, to move beyond current findings often extrapolated from IBD. This would allow for a more precise validation of the Hsp60-TLR4-NLRP3 axis as a primary driver of the persistent mucosal inflammation that remains active even during a gluten-free diet.

### Limitations and Future Perspectives

This review supports a model in which epithelial stress and innate immunity play central roles in the pathogenesis of celiac disease (CD). Heat shock proteins (Hsps), particularly Hsp27, Hsp60, and Hsp70, emerge not only as indicators of chronic cellular damage but also as active mediators capable of perpetuating the immune response through TLR activation, even in the absence of gluten. However, several limitations must be acknowledged. A primary constraint of this review is the scarcity of direct evidence linking specific Hsps to the pathogenesis of CD. Indeed, most of the mechanistic considerations proposed herein are derived from studies conducted in other chronic inflammatory bowel diseases (IBDs), such as Crohn’s disease and ulcerative colitis, where the involvement of Hsps in cellular stress responses and immune activation has been extensively documented. Although these conditions share certain inflammatory pathways and epithelial barrier alterations with CD, it must be emphasized that the extrapolation of these findings to celiac patients remains, to date, largely hypothetical. In light of these considerations, the present work does not aim to provide definitive conclusions regarding the pathogenic role of Hsps in CD, but rather to propose a possible interpretative framework supported by indirect evidence and preliminary data currently available in the literature. In this context, the hypothesis of the Hsp-TLR axis involvement in CD may represent a crucial starting point for future experimental studies. Approaches based on more physiologically relevant models, such as intestinal organoids, gut-on-a-chip systems, and immune-epithelial co-cultures, will be essential to clarify whether Hsp60 and other Hsps act as actual pathogenic mediators or simply as non-specific indicators of cellular stress.

## 5. Conclusions

In conclusion, rather than providing conclusive evidence regarding the definitive role of Hsps in CD, this review aims to stimulate further research directed at more accurately defining the molecular behavior of the chaperone system in the celiac gut. Identifying the Hsp60-TLR4 axis as a potential functional bridge between epithelial distress and innate immunity offers a novel conceptual framework that moves beyond the traditional descriptive literature. Evaluating the relevance of these proteins as biomarkers or non-canonical therapeutic targets could represent a significant step toward addressing the persistent mucosal inflammation that often characterizes celiac patients, even under a strict gluten-free diet.

## Figures and Tables

**Table 1:** Brief summary of the main members of the chaperone system and their involvement in ADs.

Family	Localization	Function	Dysregulation in ADs	Sample Size	Method
**Small** **Hsps** (HSPB1-HSPB10)	Cytosol Mitochondria Nucleus	Acts as a holdase, preventing protein aggregation by sequestering misfolded proteins [21,22].	↑ Hsp27 and αB-crystallin in **multiple sclerosis** [23].	n = 50 ctrl = 45	ELISA
			↑ HSPB8 **rheumatoid arthritis** [24].	n = 7 ctrl = 7	WB, IHC
**Hsp60** (HSP60 TRiC)	Mitochondria Cytosol	Functions as a protein foldase and prevents aggregation [25,26,27].	↑ Hsp60 in **multiple sclerosis** [28].	n = 40 ctrl = 40	ELISA Gene expression analysis
			↑ Hsp60 in **ulcerative colitis** and **Crohn’s disease** [29].	n = 40 ctrl = 20	IHC, WB, IF
			↑ IgG, IgM, and IgA autoantibodies against Hsp60 in **rheumatoid arthritis** [30].	n = 39 ctrl = 40	ELISA
**Hsp70** (SPA1A/1B HSPA1L HSPA5 HSPA9)	Cytosol Nucleus ER Mitochondria	Exhibits multiple roles in proteostasis, acting as a holdase and foldase, preventing aggregation and directing protein fate [31].	↑ HSP70 in **ulcerative colitis** at diagnosis, ↓ after therapy [32].	n = 40	IHC
			↑ IgG, IgM, and IgA autoantibodies against Hsp70 in **rheumatoid arthritis** [30].	n = 39 ctrl = 40	ELISA
			↑ HSP70 (HSP72) in **celiac disease** [33]	n = 25 ctrl = 10	WB, RT-PCR, IF
**Hsp90** (HSP90AA HSP90AB GRP9 TRAP1)	Cytosol Cytosol Cytosol/ER Mitochondria	Serves as a foldase for de novo synthesized proteins and promotes the refolding of misfolded proteins; major substrates include kinases and steroid receptors [34].	↑ Hsp90 in **multiple sclerosis** [28].	n = 40 ctrl = 40	ELISA Gene expression analysis
			↑ HSP90 in **ulcerative colitis** [32].	n = 40	IHC
			↑ IgG, IgM, and IgA autoantibodies against Hsp70 in **rheumatoid arthritis** [30].	n = 39 ctrl = 40	ELISA
**Large** **Hsps** (HSP110 GRP170)	Cytosol ER	Acts as a holdase, maintaining proteins in a non-aggregated state; functions as a co-chaperone of HSP70 [35].	No information regarding the involvement of the large HSP family in ADs was found.		

Abbreviations: ELISA, enzyme-linked immunosorbent assay; IF, immunofluorescence; IHC, immunohistochemistry; RT-PCR, reverse transcription polymerase chain reaction; WB, Western blot. n indicates the number of affected patients included in the study, while ctrl indicates healthy controls. ↑ indicates an increase in protein expression levels, whereas ↓ indicates a decrease in protein expression level.

## Data Availability

No new data were created or analyzed in this study. Data sharing is not applicable to this article.

## Abbreviations

AD	Autoimmune Disease
CD	Celiac Disease
CS	Chaperone System
DAMP	Damage-associated Molecular Patterns
EMA	Anti-endomysial Antibodies
GFD	Gluten-free Diet
HLA	Human Leukocyte Antigen
Hsps	Heat Shock Proteins
IBD	Inflammatory Bowel Disease
IELs	Intraepithelial Lymphocytes
IL-1β	Interleukin-1 beta
IL-6	Interleukin-6
MAPK	Mitogen-activated Protein Kinases
MLCK	Myosin Light Chain Kinase
NF-kB	Nuclear Factor kappa B
NLRP3	NLR Family Pyrin Domain Containing 3 (inflammasome NLRP3)
PEPs	Prolyl endopeptidases
ROS	Reactive Oxygen Species
TLRs	Toll-like Receptors
TNF- α	Tumor Necrosis Factor
tTG	Tissue Transglutaminase

## References

[1] Ilchmann-Diounou H., Menard S. (2020). Psychological stress, intestinal barrier dysfunctions, and autoimmune disorders: An overview. Front. Immunol..

[2] Pisetsky D.S. (2023). Pathogenesis of autoimmune disease. Nat. Rev. Nephrol..

[3] Fugger L., Jensen L.T., Rossjohn J. (2020). Challenges, progress, and prospects of developing therapies to treat autoimmune diseases. Cell.

[4] Marson A., Housley W.J., Hafler D.A. (2015). Genetic basis of autoimmunity. J. Clin. Investig..

[5] Vojdani A. (2014). A potential link between environmental triggers and autoimmunity. Autoimmune Dis..

[6] Tiffon C. (2018). The impact of nutrition and environmental epigenetics on human health and disease. Int. J. Mol. Sci..

[7] Song X., Liang H., Nan F., Chen W., Li J., He L., Cun Y., Li Z., Zhang W., Zhang D. (2025). Autoimmune diseases: Molecular pathogenesis and therapeutic targets. MedComm.

[8] Gujral N., Freeman H.J., Thomson A.B.R. (2012). Celiac disease: Prevalence, diagnosis, pathogenesis and treatment. World J. Gastroenterol..

[9] Ben Houmich T., Admou B. (2021). Celiac disease: Understandings in diagnostic, nutritional, and medicinal aspects. Int. J. Immunopathol. Pharmacol..

[10] Dunne M.R., Byrne G., Chirdo F., Feighery C. (2020). Coeliac disease pathogenesis: The uncertainties of a well-known immune mediated disorder. Front. Immunol..

[11] Maiuri L., Ciacci C., Ricciardelli I., Vacca L., Raia V., Auricchio S., Picard J., Osman M., Quaratino S., Londei M. (2003). Association between innate response to gliadin and activation of pathogenic T cells in coeliac disease. Lancet.

[12] Schumann M., Siegmund B., Schulzke J.D., Fromm M. (2017). Celiac disease: Role of the epithelial barrier. Cell. Mol. Gastroenterol. Hepatol..

[13] Macario A.J.L., de Macario E.C., Fink G. (2019). Chapter 12—Chaperone proteins and Chaperonopathies. Handbook of Stress, Stress Physiology, Biochemistry, and Pathology.

[14] Caio G., Volta U., Sapone A., Leffler D.A., De Giorgio R., Catassi C., Fasano A. (2019). Celiac disease: A comprehensive current review. BMC Med..

[15] Kahaly G.J., Frommer L., Schuppan D. (2018). Celiac disease and endocrine autoimmunity—The genetic link. Autoimmun. Rev..

[16] Ensari A., Marsh M.N. (2019). Diagnosing celiac disease: A critical overview. Turk. J. Gastroenterol..

[17] Cappello F., David S., Rappa F., Bucchieri F., Marasà L., Bartolotta T.E., Farina F., Zummo G. (2005). The expression of HSP60 and HSP10 in large bowel carcinomas with lymph node metastase. BMC Cancer.

[18] Caruso Bavisotto C., Cipolla C., Graceffa G., Barone R., Bucchieri F., Bulone D., Cabibi D., Campanella C., Marino Gammazza A., Pitruzzella A. (2019). Immunomorphological pattern of molecular chaperones in normal and pathological thyroid tissues and circulating exosomes: Potential use in clinics. Int. J. Mol. Sci..

[19] Zhang M., Bi X. (2024). Heat shock proteins and breast cancer. Int. J. Mol. Sci..

[20] Basset C.A., de Macario E.C., Leone L.G., Macario A.J.L., Leone A. (2023). The chaperone system in cancer therapies: Hsp90. J. Mol. Histol..

[21] Bakthisaran R., Tangirala R., Rao C.M. (2015). Small heat shock proteins: Role in cellular functions and pathology. Biochim. Biophys. Acta Proteins Proteom..

[22] Basha E., O’Neill H., Vierling E. (2012). Small heat shock proteins and α-crystallins: Dynamic proteins with flexible functions. Trends Biochem. Sci..

[23] Ce P., Erkizan O., Gedizlioglu M. (2011). Elevated HSP27 levels during attacks in patients with multiple sclerosis. Acta Neurol. Scand..

[24] Roelofs M.F., Boelens W.C., Joosten L.A.B., Abdollahi-Roodsaz S., Geurts J., Wunderink L.U., Schreurs B.W., van den Berg W.B., Radstake T.R.D.J. (2006). Identification of small heat shock protein B8 (HSP22) as a novel TLR4 ligand and potential involvement in the pathogenesis of rheumatoid arthritis. J. Immunol..

[25] Ishida R., Okamoto T., Motojima F., Kubota H., Takahashi H., Tanabe M., Oka T., Kitamura A., Kinjo M., Yoshida M. (2018). Physicochemical Properties of the Mammalian Molecular Chaperone HSP60. Int. J. Mol. Sci..

[26] Cappello F., de Macario E.C., Marasà L., Zummo G., Macario A.J.L. (2008). Hsp60 expression, new locations, functions and perspectives for cancer diagnosis and therapy. Cancer Biol. Ther..

[27] Kim H., Park J., Roh S.H. (2024). The structural basis of eukaryotic chaperonin TRiC/CCT: Action and folding. Mol. Cells.

[28] Sokolowski I., Kucharska-Lusina A., Miller E., Poplawski T., Majsterek I. (2024). Exploring the Gene Expression and Plasma Protein Levels of HSP90, HSP60, and GDNF in Multiple Sclerosis Patients and Healthy Controls. Curr. Issues Mol. Biol..

[29] Rodolico V., Tomasello G., Zerilli M., Martorana A., Pitruzzella A., Gammazza A.M., David S., Zummo G., Damiani P., Accomando S. (2010). Hsp60 and Hsp10 increase in colon mucosa of Crohn’s disease and ulcerative colitis. Cell Stress Chaperones.

[30] Mantej J., Polasik K., Piotrowska E., Tukaj S. (2019). Autoantibodies to heat shock proteins 60, 70, and 90 in patients with rheumatoid arthritis. Cell Stress Chaperones.

[31] Brocchieri L., de Macario E.C., Macario A.J. (2008). *hsp70* genes in the human genome: Conservation and differentiation patterns predict a wide array of overlapping and specialized functions. BMC Evol. Biol..

[32] Tomasello G., Sciumé C., Rappa F., Rodolico V., Zerilli M., Martorana A., Cicero G., De Luca R., Damiani P., Accardo F.M. (2011). Hsp10, Hsp70, and Hsp90 immunohistochemical levels change in ulcerative colitis after therapy. Eur. J. Histochem..

[33] Sziksz E., Veres G., Vannay Á., Prókai Á., Gál K., Ónody A., Korponay-Szabó I.R., Reusz G., Szabó A., Tulassay T. (2010). Increased heat shock protein 72 expression in celiac disease. J. Pediatr. Gastroenterol. Nutr..

[34] Hoter A., El-Sabban M.E., Naim H.Y. (2018). The HSP90 Family: Structure, Regulation, Function, and Implications in Health and Disease. Int. J. Mol. Sci..

[35] Easton D.P., Kaneko Y., Subjeck J.R. (2000). The hsp110 and Grp170 stress proteins: Newly recognized relatives of the Hsp70s. Cell Stress Chaperones.

[36] Zuo W.F., Pang Q., Zhu X., Yang Q.Q., Zhao Q., He G., Han B., Huang W. (2024). Heat shock proteins as hallmarks of cancer: Insights from molecular mechanisms to therapeutic strategies. J. Hematol. Oncol..

[37] Bukau B., Weissman J., Horwich A. (2006). Molecular chaperones and protein quality control. Cell.

[38] Samborski P., Grzymisławski M. (2015). The role of HSP70 heat shock proteins in the pathogenesis and treatment of inflammatory bowel diseases. Adv. Clin. Exp. Med..

[39] Spierings J., van Eden W. (2017). Heat shock proteins and their immunomodulatory role in inflammatory arthritis. Rheumatology.

[40] Zilaee M., Shirali S. (2016). Heat shock proteins and diabetes. Can. J. Diabetes.

[41] Gammazza A.M., Bucchieri F., Grimaldi L.M.E., Benigno A., de Macario E.C., Macario A.J.L., Zummo G., Cappello F. (2012). The molecular anatomy of human Hsp60 and its similarity with that of bacterial orthologs and acetylcholine receptor reveal a potential pathogenetic role of anti-chaperonin immunity in myasthenia gravis. Cell. Mol. Neurobiol..

[42] Hoter A., Naim H.Y. (2019). The functions and therapeutic potential of heat shock proteins in inflammatory bowel disease—An update. Int. J. Mol. Sci..

[43] Kostenko S., Moens U. (2009). Heat shock protein 27 phosphorylation: Kinases, phosphatases, functions and pathology. Cell. Mol. Life Sci..

[44] Paul C., Manero F., Gonin S., Kretz-Remy C., Virot S., Arrigo A.P. (2002). Hsp27 as a negative regulator of cytochrome C release. Mol. Cell. Biol..

[45] Setty M., Discepolo V., Abadie V., Kamhawi S., Mayassi T., Kent A., Ciszewski C., Maglio M., Kistner E., Bhagat G. (2015). Distinct and synergistic contributions of epithelial stress and adaptive immunity to functions of intraepithelial killer cells and active celiac disease. Gastroenterology.

[46] Bavisotto C.C., Alberti G., Vitale A.M., Paladino L., Campanella C., Rappa F., Gorska M., de Macario E.C., Cappello F., Macario A.J.L. (2020). Hsp60 post-translational modifications: Functional and pathological consequences. Front. Mol. Biosci..

[47] Hu C., Yang J., Qi Z., Wu H., Wang B., Zou F., Mei H., Liu J., Wang W., Liu Q. (2022). Heat shock proteins: Biological functions, pathological roles, and therapeutic opportunities. MedComm.

[48] Piatek-Guziewicz A., Ptak-Belowska A., Przybylska-Felus M., Pasko P., Zagrodzki P., Brzozowski T., Mach T., Zwolinska-Wcislo M. (2017). Intestinal parameters of oxidative imbalance in celiac adults with extraintestinal manifestations. World J. Gastroenterol..

[49] Barone M.V., Auricchio R., Nanayakkara M., Greco L., Troncone R., Auricchio S. (2022). Pivotal role of inflammation in celiac disease. Int. J. Mol. Sci..

[50] Calderwood S.K., Gong J., Murshid A. (2016). Extracellular HSPs: The complicated roles of extracellular HSPs in immunity. Front. Immunol..

[51] Zininga T., Ramatsui L., Shonhai A. (2018). Heat shock proteins as immunomodulants. Molecules.

[52] Quintana F.J., Cohen I.R. (2011). The HSP60 immune system network. Trends Immunol..

[53] Swaroop S., Sengupta N., Suryawanshi A.R., Adlakha Y.K., Basu A. (2016). HSP60 plays a regulatory role in IL-1β-induced microglial inflammation via TLR4-p38 MAPK axis. J. Neuroinflamm..

[54] Swaroop S., Mahadevan A., Shankar S.K., Adlakha Y.K., Basu A. (2018). HSP60 critically regulates endogenous IL-1β production in activated microglia by stimulating NLRP3 inflammasome pathway. J. Neuroinflamm..

[55] Zhen Y., Zhang H. (2019). NLRP3 inflammasome and inflammatory bowel disease. Front. Immunol..

[56] Li Z., Guo J., Bi L. (2020). Role of the NLRP3 inflammasome in autoimmune diseases. Biomed. Pharmacother..

[57] Al-Sadi R., Ye D., Said H.M., Ma T.Y. (2010). IL-1beta-induced increase in intestinal epithelial tight junction permeability is mediated by MEKK-1 activation of canonical NF-kappaB pathway. Am. J. Pathol..

[58] Kaminsky L.W., Al-Sadi R., Ma T.Y. (2021). IL-1β and the intestinal epithelial tight junction barrier. Front. Immunol..

[59] Sturgeon C., Fasano A. (2016). Zonulin, a regulator of epithelial and endothelial barrier functions, and its involvement in chronic inflammatory diseases. Tissue Barriers.

[60] Szebeni B., Veres G., Dezsofi A., Rusai K., Vannay A., Bokodi G., Vásárhelyi B., Korponay-Szabó I.R., Tulassay T., Arató A. (2007). Increased mucosal expression of Toll-like receptor (TLR)2 and TLR4 in celiac disease. J. Pediatr. Gastroenterol. Nutr..

[61] Talipova D., Smagulova A., Poddighe D. (2022). Toll-like receptors and celiac disease. Int. J. Mol. Sci..

[62] Vabulas R.M., Ahmad-Nejad P., da Costa C., Miethke T., Kirschning C.J., Häcker H., Wagner H. (2001). Endocytotic HSP60s use the toll-like receptor 2 (TLR2) and TLR4 to activate the toll/interleukin-1 receptor signaling pathway in innate immune cells. J. Biol. Chem..

